# Impact of a Rapid Change in Beta-lactam Resistance Epidemiology on the Management of Patients With Bloodstream Infections

**DOI:** 10.1093/ofid/ofag225

**Published:** 2026-04-29

**Authors:** Laura Bobbitt, Lili Tao, Benjamin Ereshefsky, Austin Ing, Romney Humphries

**Affiliations:** Department of Pharmaceutical Services, Vanderbilt University Medical Center, Nashville, Tennessee, USA; Department of Pathology, Microbiology and Immunology, Vanderbilt University Medical Center, Nashville, Tennessee, USA; Department of Pharmaceutical Services, Vanderbilt University Medical Center, Nashville, Tennessee, USA; Department of Pharmaceutical Services, Vanderbilt University Medical Center, Nashville, Tennessee, USA; Department of Pathology, Microbiology and Immunology, Vanderbilt University Medical Center, Nashville, Tennessee, USA

**Keywords:** CTX-M, bacteremia, PCR, E. coli, K. pneumoniae

## Abstract

The impact of a shift in the epidemiology of ceftriaxone resistance among *Escherichia coli* and *Klebsiella pneumoniae* recovered from blood cultures was evaluated. 163 patients were included for analysis, 139 (85.3%) with *bla*_CTX-M_ detected and 24 (14.7%) without *bla*_CTX-M_ detected by molecular testing. All but 1 case in the no-CTX-M cohort occurred in 2024. Patients in the *bla*_CTX-M_ detected cohort were started on optimal antimicrobial treatment significantly earlier than patients in the no *bla*_CTX-M_ detected group (18.6 vs 59.2 hours, *P* < .001). Between 2022 and 2024, the rate of CTX-M negative ceftriaxone-resistant *E. coli* (*n* = 14) and *K. pneumoniae* (*n* = 10) increased from 3.2% to 5.6% to 20.3%, while at the same time the rate of ceftriaxone susceptibility decreased for both species. Whole genome sequencing identified AmpC genes (*bla*_CMY-2_, *bla*_DHA-1_, and *bla*_FOX-5_), TEM extended-spectrum beta-lactamases (ESBL) variants (*bla*_TEM-190_) and SHV ESBL variants (*bla*_SHV-12_ and *bla*_SHV-7_) as the likely cause of ceftriaxone resistance in these isolates. Despite this change, the negative predictive value for the test was 94% in 2024. Our data suggest continued reliance on molecular testing for de-escalation is appropriate for most patients, although rapid follow-up of final susceptibility results is warranted. In addition, institutions should be aware that epidemiology changes may occur and routinely monitor the incidence of ceftriaxone resistant but *bla*_CTX-M_-negative isolates.

Rapid initiation of effective antimicrobial therapy is a cornerstone for optimizing sepsis outcomes [1]. Many institutions utilize cefepime or piperacillin-tazobactam as preferred empirical coverage for Gram-negative bacteria in patients who present with sepsis, while awaiting blood culture results [2]. However, resistance to broad-spectrum cephalosporins, including cefepime, is increasingly common among *Escherichia coli* and *Klebsiella pneumoniae*, the two Gram-negative bacteria most commonly associated with sepsis in the United States, due to the expansion of extended-spectrum beta-lactamases (ESBLs) in these two species [3, 4]. Furthermore, data indicate that the risk of treatment failure is significantly higher when piperacillin-tazobactam is used to treat ceftriaxone-resistant *E. coli* or *K. pneumoniae,* as compared to meropenem [5]. Based in part on these data, the Infectious Diseases Society of America treatment guidelines for antimicrobial resistant Gram-negative bacterial infections suggest carbapenems as the preferred antimicrobial agents for treating ESBL-producing *E. coli* and *K. pneumoniae* infections outside the urinary tract [6]. Thus, there is an increasing need for rapid diagnostic tests to detect ESBLs in blood cultures.

Time to full identification and susceptibility results for bacteria isolated from blood cultures, using standard approaches, is a median of 2.7 days in U.S. laboratories [7]. The time to identification of antimicrobial resistance in these cultures can be shortened using multiplexed molecular panels, which detect key resistance genes. Commercially available, U.S. Food and Drug Administration-cleared multiplexed blood culture panels for Gram-negative bacteria include detection of selected carbapenemase genes (ie, *bla*_KPC_, *bla*_NDM_, *bla*_IMP_, *bla*_VIM_, and *bla*_OXA-48-like_) and the ESBL gene family, *bla*_CTX-M_ [8–10]. Retrospective studies showed earlier detection of *bla*_CTX-M_ using these panels is associated with reduced time to treatment with a carbapenem [11]. A recent meta-analysis similarly showed that when these tests are combined with antibiotic stewardship action, a significant reduction in time to optimal antimicrobial therapy and reduction in mortality for patients with bacteremia can be achieved [12]. Furthermore, the negative predictive value (NPV) of *bla*_CTX-M_ gene detection for ceftriaxone resistance in *E. coli* and *K. pneumoniae* is ∼95% in most hospitals, enabling confident de-escalation to ceftriaxone in cases where *bla*_CTX-M_ result is negative [13].

The high NPV of CTX-M for ceftriaxone resistance is driven by the fact that *bla*_CTX-M_ is the most common resistance mechanism among ceftriaxone-resistant *E. coli* and *K. pneumoniae* in the United States. However, ceftriaxone-resistance mechanisms are regional, and 12–50% of ceftriaxone resistance in *E. coli* and *K. pneumoniae* is caused by other resistance mechanisms, including TEM or SHV ESBLs or acquired AmpC enzymes [14]. The present study evaluated the clinical impact of a sudden shift in the epidemiology of ceftriaxone-resistant *E. coli* and *K. pneumoniae* at a large academic medical center, from predominantly CTX-M to non-CTX-M resistance mechanisms.

## METHODS

### Study Setting and Design

This study was conducted at Vanderbilt University Medical Center, which includes a large academic teaching hospital, a stand-alone children's hospital, and three regional community hospitals. All microbiology testing for the system is performed at a centralized location.

All patients with ceftriaxone-resistant *E coli* or *K pneumoniae* isolated from blood cultures between January 1, 2022 and August 31, 2024 were included in this study. Exclusion criteria included patients with polymicrobial blood cultures, death within 48 hours of index culture collection, treatment at an outside hospital, and patients with carbapenem-resistant *E. coli* or *K. pneumoniae* bacteremia.

Patient cohorts were divided into those for whom *bla*_CTX-M_ was detected by molecular testing and those where *bla*_CTX-M_ was not detected. The following antimicrobial stewardship measures were performed for patients with positive blood cultures throughout the study: infectious diseases pharmacists reviewed positive blood cultures every weekday and select weekends to ensure optimized antimicrobial regimens. When molecular results were available and susceptibilities were pending, the pharmacists optimized the antimicrobial regimen based on presence of *bla*_CTX-M_, historical culture results, comorbidities, allergies, drug interactions, and other patient-specific factors. Once susceptibilities resulted, pharmacists further tailored antimicrobial regimens based on available data. In the setting of ceftriaxone resistance without detectable *bla_CTX_*_-M_ on multiplex molecular panel, pharmacists ensured that ceftriaxone was discontinued and an effective agent was initiated.

### Microbiology

Blood cultures were performed as part of standard of care, using a Bactec (BD, Sparks, MD) system and BACTEC Lytic Anaerobic and Plus Aerobic bottles. Upon flagging positive, gram stain was performed and reported via an electronic alerts system. Blood cultures demonstrating Gram-negative bacteria were tested using the cobas® eplex BCID-GN (Roche, Indianapolis, IN) and results of this testing were also sent via the alert system. Antimicrobial susceptibility testing was performed the following day from colony growth of subcultured blood culture broth using the Phoenix M50 automated microbiology system and NMIC-306 panels (BD, Sparks, MD, from January 1, 2022 to March 1, 2024) or Vitek2 (bioMérieux, Durham, NC, from March 1, 2024 to August 31, 2024).

Reference broth microdilution (BMD) using panels prepared in-house was performed prospectively for isolates that were negative for the *bla*_CTX-M_ target and ceftriaxone resistant by standard of care methods. BMD was performed as described previously [15]. Retrospectively, available isolates were sequenced; 15/24 isolates were available for this analysis. Bacterial genomic DNA was extracted from the isolates and whole genome sequencing was performed using Illumina NovaSeq (Illumina, CA). Genomic assembly and annotation were performed using the pipelines available from the Bacterial and Viral Bioinformatics Resource Center (BV-BRC, https://www.bv-brc.org/). Antimicrobial resistance genes for each isolate were identified on BV-BRC using National Database of Antibiotic Resistant Organisms as the data source.

Antibiogram data were generated using WHONET. A single isolate per patient per calendar year was used in calculations, according to CLSI M39 standards [16].

### Study Measures

All data were abstracted manually from the electronic medical record through chart review by a study team member. Time to optimal antimicrobial therapy was calculated from the time of blood culture collection. Clinical outcomes such as length of hospitalization or death were not evaluated, due to the heterogenous population included in this study. Pitt bacteremia scores were calculated as described elsewhere [17].

### Patient Consent Statement

This study was approved by the Vanderbilt University Medical Center Institutional Review Board with waiver of consent as a quality improvement project.

## RESULTS

The rate of ceftriaxone susceptibility among *E. coli* isolated from blood cultures was significantly lower in 2024 (*n* = 375 patients, 75.6%, 95% CI, 71.3–79.9%) than in 2023 (*n* = 379 patients, 81.4% [95% CI, 76.6–86.2% – 88.1%], *P* = .0027), or 2022 (*n* = 249 patients, 84.4% [95% CI, 80.7–88.1%], *P* = .007). Similarly, ceftriaxone susceptibility was numerically lower among blood isolates of *K. pneumoniae* in 2024 (*n* = 205 patients, 70%, 95% CI, 63.7–76.3%) than in 2023 (*n* = 194 patients, 79.5%, 95% CI, 73.8–85.2%) or 2022 (*n* = 122 patients, 78.7%, 95% CI, 71.4–86.0%), although these differences did not achieve statistical significance. The proportion of ceftriaxone-resistant blood stream *E. coli* and *K. pneumoniae* isolates for which *bla*_CTX-M_ was not detected was significantly higher in 2024 (20.3%) than in 2023 (5.6%, *P* = .01) and 2022 (3.2%, *P* = .002). The negative predictive value of *bla*_CTX-M_ detection for ceftriaxone resistance was 94.3% (95% CI, 90.8–96.6%), 99.2% (95% CI, 99.3–99.9%) and 99.5% (95% CI, 97.8–99.9%) in 2024, 2023, and 2022, respectively. The positive predictive value of *bla*_CTX-M_ detection for ceftriaxone resistance was 100% across all three study years. All ceftriaxone resistance was confirmed by BMD for the isolates that were ceftriaxone-resistant but *bla*_CTX-M_ negative.

A total of 193 patients with ceftriaxone-resistant *E. coli* or *K. pneumoniae* bacteremia were identified from January 2022 to August 2024. Thirty patients were excluded from analysis, including 18 with polymicrobial bacteremia, 7 who died within 48 hours of culture collection, 3 with carbapenem-resistant Enterobacterales (CRE), and 2 treated at an outside hospital. The remaining 163 patients were included for analysis, 139 (85.3%) with *bla*_CTX-M_ detected and 24 (14.7%) without *bla*_CTX-M_ detected by ePlex. Table 1 shows general demographic and clinical characteristics. The median age was 62.4 years (range, 52.6–68.8). Sixty-one (45.2%) patients had underlying diabetes and 59 (43.7%) had cancer. The most common underlying source of bacteremia was the urinary tract (49.7%), followed by intra-abdominal (29.4%). The bloodstream infections were classified as community-acquired in 116 patients (71.2%), defined as index blood culture collected <48 hours after presentation to the hospital. A total of 53 patients (32.5%) were admitted to the intensive care unit (ICU) within 72 hours of index blood culture collection.

**Table 1.** Demographic and Clinical Characteristics

	CTX-M Detected (*n* = 139)	CTX-M Not Detected (*n* = 24)	Total (*N* = 163)
Age, y	62.8 (52.9–69.2)	61.0 (51.7–66.0)	62.4 (52.6–68.8)
Female	64 (46%)	11 (45.8%)	75 (46%)
Comorbidities			
Diabetes mellitus	50 (44.2%)	11 (50%)	61 (45.2%)
End stage renal disease	14 (12.4%)	3 (13.6%)	17 (12.6%)
Liver cirrhosis	11 (9.7%)	2 (9.1%)	13 (9.6%)
Cancer	48 (42.5%)	11 (50%)	59 (43.7%)
Solid organ transplant	32 (28.3%)	7 (31.8%)	39 (28.9%)
Stem cell transplant	23 (20.4%)	2 (9.1%)	25 (18.5%)
Neutropenia	27 (23.9%)	2 (9.1%)	29 (21.5%)
Source of bacteremia			
UTI	66 (47.5%)	15 (62.5%)	81 (49.7%)
Intra-abdominal	41 (29.5%)	7 (29.2%)	48 (29.4%)
Unknown	16 (11.5%)	1 (4.2%)	17 (10.4%)
Respiratory	6 (4.3%)	0	6 (3.7%)
Central line	5 (3.6%)	1 (4.2%)	6 (3.7%)
SSTI	3 (2.2%)	0	3 (1.8%)
Other	2 (1.4%)	0	2 (1.2%)
Timing of bacteremia			
Community	98 (70.5%)	18 (75%)	116 (71.2%)
Hospital	41 (29.5%)	6 (25%)	47 (28.8%)
ICU admission	42 (30.2%)	11 (45.8%)	53 (32.5%)
Pitt bacteremia score			
Median	1 (0–3)	2 (0–3)	1 (0–3)
*N* ≥4	18 (12.9%)	4 (16.7%)	22 (13.5%)

Antimicrobial utilization is displayed in Table 2. Cefepime was the most common empiric antimicrobial agent prescribed in the *bla*_CTX-M_ detected group (43.2%) whereas piperacillin-tazobactam was more common in the no *bla*_CTX-M_ group (70.8%). Patients in the *bla*_CTX-M_ detected group were started on optimal antimicrobial treatment significantly earlier than patients in the no *bla*_CTX-M_ detected group (18.6 vs 59.2 hours, *P* < .001). The median duration of optimal therapy did not differ significantly between cohorts (14 vs 11.6 days, *P* = .251).

**Table 2.** Organism Data and Antibiotic Utilization

	CTX-M Detected (*n* = 139)	CTX-M Not Detected (*n* = 24)	Total (*N* = 163)	*P* value
Organism				.828
*E. coli*	77 (55.4%)	14 (58.3%)	91 (55.8%)	
*K. pneumoniae*	62 (44.6%)	10 (41.7%)	72 (44.2%)	
Cefepime MIC (interpretation)				<.001
≤2 µg/mL (S)	13 (9.4%)	12 (50%)	25 (15.3%)	
4–8 µg/mL (SDD)	24 (17.2%)	3 (12.5%)	27 (16.6%)	
≥16 µg/mL (R)	102 (73.4%)	9 (37.5%)	111 (68.1%)	
Piperacillin-tazobactam MIC (interpretation)				.660
≤8 µg/mL (S)	80 (57.6%)	16 (66.7%)	96 (58.9%)	
16 µg/mL (SDD)	28 (20.1%)	3 (12.5%)	31 (19.0%)	
≥32 µg/mL (R)	31 (22.3%)	5 (20.8%)	36 (22.1%)	
Empiric antimicrobial				.003
Ceftriaxone	17 (12.2%)	0	17 (10.4%)	
Cefepime	60 (43.2%)	6 (25%)	66 (40.5%)	
Piperacillin-tazobactam	44 (31.7%)	17 (70.8%)	61 (37.4%)	
Meropenem	15 (10.8%)	0	15 (9.2%)	
Fluoroquinolone	3 (2.2%)	1 (4.2%)	4 (2.5%)	
Time to optimal treatment, h	18.6 (15.9–24.2)	59.2 (44.0–85.7)	19.0 (16.2–28.3)	<.001
Definitive treatment with carbapenem	124 (89.2%)	19 (79.2%)	143 (87.7%)	.180
Duration of treatment with optimal antibiotic, d	14 (8.7–15.8)	11.6 (8.8–14.5)	13.8 (8.7–15.7)	.251

Patients in the no *bla*_CTX-M_ group were evaluated for de-escalation to ceftriaxone based on the ePlex results. Two patients had empirical therapy narrowed to ceftriaxone following ePlex results. The first was a patient with metastatic lung cancer who had been started on empirical cefepime. Cefepime was de-escalated to ceftriaxone in response to the ePlex results (*E. coli* that was CTX-M negative). The patient was escalated to meropenem once final susceptibility results were reported, 55 hours after the index culture. This patient was transitioned to palliative care and expired 6 days later. The second patient was newly diagnosed with HIV and disseminated histoplasmosis, who was admitted to the medical ICU for sepsis. This patient similarly was de-escalated to ceftriaxone after ePlex results (CTX-M negative *E. coli*) were reported but escalated to ertapenem when an *E. coli* recovered from urine cultures was reported as ceftriaxone-resistant, 55 hours after the index blood culture. This patient was stabilized and transitioned out of the ICU 3 days later.

Fifteen isolates that were ceftriaxone-resistant, but *bla*_CTX-M_ negative were available for further analysis, representing 62.5% of the CTX-M negative cases presented herein. Whole genome sequencing was performed on these isolates, which identified AmpC genes (*bla*_CMY-2_, *bla*_DHA-1_, and *bla*_FOX-5_), TEM ESBL variants (*bla*_TEM-190_) and SHV ESBL variants (*bla*_SHV-12_ and *bla*_SHV-7_) as the likely cause of ceftriaxone resistance in these isolates (Table 3). Several *E. coli* isolates harbored variants of the *E. coli* chromosomal AmpC gene, *bla*_EC_ [18]. One *E. coli* isolate was found to be positive for *bla_CTX_*_-M-15_ by sequencing, but negative by the ePlex. No isolates were clonally related (not shown). In this subset of isolates, 7 of 8 isolates with an ESBL were susceptible to cefepime (Table 3) and 5 of 7 with no ESBL detected were susceptible to cefepime.

**Table 3.** Microbiology Test Results for Isolates Found to be Ceftriaxone-resistant but Negative for blaCTX-M by ePlex

Year	Organism	Beta-lactamase Genes Detected By WGS			MIC (µg/mL)^a^		
		AmpC-Type	ESBL-Type	Narrow-spectrum	CRO	FEP	TZP
2023	*E. coli*	*bla*_CMY-2_, *bla*_EC-1_	None	*bla*_TEM-1_	**8**	≤.5	≤4
2024	*E. coli*	*bla*_EC-5_	*bla*_SHV-12_	*bla*_TEM-1_, *bla*_LPA-1_	**8**	≤0.12	≤4
2024	*E. coli*	*bla*_CMY-2_, *bla*_EC-8_	None	None	**32**	≤0.12	≤4
2024	*E. coli*	*bla*_DHA-1_, *bla*_EC-8_	None	None	**32**	**16**	**>64**
2024	*E. coli*	*bla*_CMY-2_, *bla*_EC-13_, *bla*_DHA-1_	None	None	**32**	0.5	**>64**
2024	*E. coli*	*bla*_EC-5_	bla_CTX-M-15_	*bla*_OXA-1_	**>32**	**16**	8
2024	*E. coli*	None	bla_TEM-190_	None	**4**	2	>**64**
2024	*E. coli*	*bla*_CMY-2_	None	None	**32**	≤0.12	8
2024	*E. coli*	None	bla_SHV-12_	*bla*_TEM-1_	**32**	4	≤4
2024	*K. pneumoniae*	*bla*_FOX-5_	None	*bla*_CARB-2_, *bla*_SHV-1_	**32**	2	8
2024	*K. pneumoniae*	None	bla_SHV-7_	*bla*_OKP-B-3_	**32**	≤0.12	≤4
2024	*K. pneumoniae*	*bla*_FOX-5_	None	*bla*_CARB-2_, *bla*_SHV-1_	**>32**	**>16**	16
2024	*K. pneumoniae*	None	bla_SVH-12_	*bla*_LPA-1_, *bla*_LEN-2_, *bla*_TEM-1_	**32**	1	≤4
2024	*K. pneumoniae*	None	bla_SVH-12_	*bla*_LPA-1_, *bla*_LEN-2_, *bla*_TEM-1_	**32**	1	8
2024	*K. pneumoniae*	None	bla_SHV-12_	*bla*_TEM-1_	**32**	≤0.12	≤4

^a^Bold values indicate resistance by 2025 CLSI M100 standards.

## DISCUSSION

Bacterial resistance mechanisms are dynamic and may shift over relatively short periods. For example, a 461% increase in NDM-producing *Enterobacterales* was recently reported by the Centers for Disease Control and Prevention over a 5-year period [19]. In general, however, major changes are uncommon year over year in the antibiogram. We observed both an increase in ceftriaxone resistance and in the epidemiology of the resistance mechanisms for ceftriaxone resistance, over a relatively short period of time (6 months). This shift was initially identified by the antimicrobial stewardship pharmacists when a drug-bug mismatch was found in treatment. A subsequent systematic review of the epidemiology at our institution found that 1 in 5 cases of ceftriaxone-resistance in *E. coli* and *K. pneumoniae* were in *bla*_CTX-M_ negative isolates, in contrast to a historical 3–5% seen, both at our institution, and by others [3, 20, 21]. The rapid, global dissemination of CTX-M is thought to be due to both the relative ease of mobilization of *bla*_CTX-M_ genes to virtually all major plasmid incompatibility groups circulating in Enterobacterales, and the association of CTX-M-encoding plasmids with successful virulent clonal lineages of *E. coli* and *K. pneumoniae*, such as *E. coli* ST131 [22]. In contrast, the shift in epidemiology at our institution was not due to the outbreak of a given strain type, or even the clonal expansion of a given resistance mechanism (Table 2). Thus, it is unclear why this change happened at such a rapid rate, or if this shift represents a sporadic anomaly in the typical epidemiology of ceftriaxone resistance.

Patients with *bla*_CTX-M_ not detected were more often started on piperacillin-tazobactam therapy empirically (Table 1). Patients with ceftriaxone-resistant infections and no *bla*_CTX-M_ detected had substantially longer times (average, 40.6 hours more) to optimal therapy (Table 2). Piperacillin-tazobactam resistance is mainly related to expression of OXA-1 [23], a tazobactam-resistant penicillinase that is typically co-carried with CTX-M ESBLs [3]. In the MERINO trial, 86% of ceftriaxone nonsusceptible isolates harbored CTX-M, and isolates with both OXA-1 and an ESBL had higher piperacillin-tazobactam MICs than those with ESBLs alone [24]. Detection of OXA-1 phenotypically through piperacillin-tazobactam MIC testing is challenging, if not impossible [25], and the presence of CTX-M may serve as a reasonable surrogate for likelihood of OXA-1 presence. Indeed, in our cohort, the only isolate in the no-CTX-M cohort with *bla*_OXA-1_ detected by whole genome sequencing was the isolate with a false-negative *bla*_CTX-M_ result (Table 3). As such, it is possible that the risk of not rapidly escalating from piperacillin-tazobactam to a carbapenem if CTX-M is not detected is lower than if CTX-M is detected, as these isolates are less likely to harbor OXA-1. Similarly, isolates without CTX-M in our cohort were more likely to be cefepime-susceptible, and others have shown non-CTX-M ESBL treatment success with cefepime, albeit the case number was exceedingly small (*N* = 5). This hypothesis, however, needs validation with much larger data sets, which poses many challenges as the mechanism of ceftriaxone resistance is rarely characterized in observational studies or clinical trials [26].

Our study has some limitations. First, we did not evaluate patients with ceftriaxone-susceptible infections. In addition, we did not evaluate clinical outcomes due to the heterogenous patient population and low number of CTX-M negative patients in this study. We did not have access to the isolates from all patients included in this study for genome sequencing, although all were prospectively tested for ceftriaxone resistance by reference methods.

Overall, the global prevalence of ESBL production by *E. coli* and *K. pneumoniae* has increased to >20% over the past 20 years [27]. Greater than 97% of ESBL producers harbor *bla*_CTX-M_ [28], which underscores the value of detecting this resistance mechanism on molecular panels. In our study, we encountered a single case of false-negative *bla*_CTX-M_ detection, which reflects the excellent sensitivity for detection of this gene by molecular panels [29]. The negative predictive value for ceftriaxone resistance when *bla*_CTX-M_ was not detected remained high at 94.3%, despite the relatively low sensitivity for ceftriaxone resistance (79.7%) at our institution in 2024. Together, these data suggest continued reliance on molecular testing for de-escalation is appropriate for most patients, although rapid follow-up of final susceptibility results is warranted. In addition, institutions should be aware that epidemiology changes may occur and routinely monitor the incidence of ceftriaxone-resistant but *bla*_CTX-M_-negative isolates. In the future, next generation molecular blood culture panels will include additional resistance markers [15], such as TEM and SHV ESBL and AmpC genes, which will further help detection of these epidemiological anomalies in real-time.
